# Burn out of health care professionnels leads to addiction

**DOI:** 10.1192/j.eurpsy.2023.1000

**Published:** 2023-07-19

**Authors:** S. Elkardi, H. Choujaa, M. Agoub

**Affiliations:** psychiatry, chu ibn rochd, casablanca, Morocco

## Abstract

**Introduction:**

Despite their knowledge about the risks and treatment options for substance abuse disorders, healthcare workers are not immune to them. Meanwhile, a number of studies have shown that health professionals have an increased risk of depression, addictive diseases and burnout due to the occupation-linked mental and physical burden and in particular an increased prevalence of substance-related disorders.

**Objectives:**

The aim of our present study was to focus on prevalence and the risk factors of addiction behavior and its possible association with burnout among healthcare workers in a moroccan hospital applying a questionnaire-based survey.

**Methods:**

Questionnaires were distributed to 250 healthcare workers of CHU IBN ROCHD Casablanca, Morocco. A total of 200 participants completed the survey. Data collected in 2021 were analysed by using descriptive statistics, an independent t test and Pearson’s correlation analysis.

**Results:**

In our sample, the 26-35 year old age group presents 74%, 79% of the participants are women, 38.5% of the participants have freelance status, 62.5% are single, 78.8% of participants have no children, 60% of the participants are residents and 33.2% are nurses. Almost half of the participants have between 1 and 5 years of training or work experience. 37.2% reported a history of an anxiety disorder and 33.1% have a history of a depressive disorder. 90.2% have never used any substance.

45% spend more than 3 hours, 86.6% of the participants used the internet between 6pm and midnight.

In total, 20.5% suffered from mild burnout, 31% moderate burnout and 48.5% severe burnout, according to the Maslach Burnout Inventory. The prevalence of depression was 32%; that of anxiety was 29.5%.

Factors associated with Burn-out in uni-variate study were: age group (p = 0.044) ,marital status (p= 0.001), preferred time of connection (p = 0.019), burnout (p=0.001), depersonalization (p=0.001), personal fulfillment (p=0.001), internet use for work (p = 0.0019), internet use for leisure ( p = 0.002) and also for online games (p = 0.016).

Factors associated with depersonalization were marital status (p=0.001), number of children ( p = 0.042), psychiatric history (p=0.001), substance abuse ( p = 0.012), preferred time of day to use the internet (p = 0.001), use of the internet for social networking (p =0.03), online gaming (p = 0.008).

Factors associated with personal accomplishment were age (p=0.001), number of children (p=0.016), use of the internet for work (p=0.001).

**Conclusions:**

A significant proportion of our healthcare workers suffered from burnout, depression and anxiety disorders, which was associated with substance and internet abuse in univariate analysis. Our study also draws attention to the risk factors of burnout such as age, family status, working type and working hours internet use, substance use. The possible association of burnout and other addiction behaviors merits further investigation.

**Disclosure of Interest:**

None Declared

